# Investigations of CrN/TiO_2_ Coatings Obtained in the Hybrid PVD/ALD Process on 316L Steel Substrates

**DOI:** 10.3390/ma19101921

**Published:** 2026-05-07

**Authors:** Marcin Staszuk, Daniel Pakuła, Łukasz Reimann, Anna Woźniak, Anna Kloc-Ptaszna, Julia Kolasa, Paweł Nuckowski

**Affiliations:** 1 Faculty of Mechanical Engineering, Silesian University of Technology, Konarskiego St. 18a, 44-100 Gliwice, Poland; daniel.pakula@polsl.pl (D.P.); lukasz.reimann@polsl.pl (Ł.R.); anna.wozniak@polsl.pl (A.W.); anna.kloc@polsl.pl (A.K.-P.); pawel.nuckowski@polsl.pl (P.N.); 2 Faculty of Biomedical Engineering, Silesian University of Technology, Roosevelta 40, 41-800 Zabrze, Poland; julia.kolasa@polsl.pl

**Keywords:** PVD, ALD, hybrid coatings, 316L steel, corrosion resistance, tribology

## Abstract

Chromium nitride (CrN) can be used as a coating material deposited via physical vapour deposition (PVD), thereby improving the corrosion and wear resistance of the substrate. However, this level of corrosion protection may not be sufficient in an aggressive corrosion environment. The coatings often contain intrinsic microstructural defects, such as microcraters, which can serve as pathways for the corrosive medium to reach the substrate, thereby initiating and promoting corrosion. In this study, the influence of parameters on the formation of a TiO_2_ layer using the ALD technique was investigated. In particular, the work focused on assessing the effectiveness of the TiO_2_ layer as a sealing barrier for CrN coatings (PVD) applied to austenitic 316L steel. The TiO_2_ ALD coatings were produced at a constant temperature of 200 °C with a varying number of cycles, ranging from 200 to 1000 cycles. Structural investigations were carried out using scanning electron microscopy SEM and atomic force microscopy. Electrochemical properties were investigated using a potentiodynamic test and electrochemical impedance spectroscopy (EIS) in a 3.5% NaCl solution. SEM observations indicate that the morphology of the hybrid coatings is strongly influenced by the number of ALD cycles. The TiO_2_ layer conformally reproduces the underlying PVD topography while progressively sealing the coating by filling intrinsic defects and discontinuities. Hybrid coatings (PVD/ALD) with titanium oxide deposited at 500 ALD cycles were found to have the best corrosion resistance. The polarisation resistance for these coatings was nearly four times higher than that of both the single PVD (CrN) coating and the uncoated stainless steel 316L substrate. At the same time, the corrosion current density was several times lower than that of the reference systems. The corrosion mechanisms were investigated by observing the surfaces of the samples after corrosion testing using SEM. Abrasion resistance tests using the pin-on-disc method and adhesion tests (scratch tests) were also performed, which showed that appropriate optimisation of the layer architecture in the PVD/ALD hybrid system significantly improves its tribological durability, interlayer stability, and adhesion to the substrate.

## 1. Introduction

Over the past few years, there has been an ongoing search for optimal, biocompatible, nonallergenic materials for bone and joint implants with high durability and quality. Among the many materials used primarily for bone stabilisers (screws and plates) or joint endoprostheses (hip and knee implants), austenitic 316L stainless steel is the most widely used for economic reasons, as it offers a favourable quality-to-cost ratio combined with good corrosion resistance and well-established clinical performance, making it a reliable and cost-effective solution, particularly in high-volume orthopaedic applications. In comparison, the preference for stainless steel in short-term implants, as opposed to titanium, is primarily attributed to the higher production and processing costs associated with titanium compared to more commonly used metals such as stainless steel. However, when stainless steel is not subjected to appropriate surface treatment, its presence inside the human body may induce an allergic reaction. Allergic reactions associated with this material are primarily caused by nickel, which is an indispensable alloying element of austenitic stainless steels. To prevent such adverse effects and simultaneously improve antibacterial and osteointegration properties, appropriate surface treatment modification techniques are applied [1,2,3,4,5,6,7,8,9,10]. It has been proven in a number of research works [11,12,13,14,15,16,17,18,19,20,21,22,23] that the best treatment in this case is the use of a hybrid surface treatment, consisting of two technological processes: the physical vapour deposition (PVD) process and the ALD atomic layer deposition process (Table 1). The first method (PVD) allows for the production of layers with optimal thickness, high mechanical or fatigue strength, and corrosion resistance. The most common coating materials obtained by this technique include the following phases: TiN, CrN, TiO_2_, AlCrTiSiN, TiSiN, AlN, AlCrN, CrAlN, and TiAlSiN. Due to the high hardness of these coatings and high abrasion resistance, they can operate under complex mechanical loads. Furthermore, they are characterised by high corrosion resistance. Unfortunately, the peculiarities of the processes by which these coatings are produced cause discontinuities or craters to form on the surface in their crystalline structure. These defects can affect the functional properties of these materials. Therefore, to prevent deterioration of properties, thin ALD layers are additionally produced in a second technological process. The task of these layers (as proved in earlier research work) is to seal PVD coatings. The most commonly used sealing (barrier) layers mainly include the oxides TiO_2_, ZnO, Al_2_O_3_, and Ta_2_O_5_. PVD + ALD hybrid systems can be built as follows: first, barriers of ALD layers are applied between PVD layers; second, ALD sealing coatings are applied on PVD coatings. In the first case, mechanical properties are improved in particular, while corrosion resistance is improved in the second case. The ALD process, characterized by sequential, self-limiting surface reactions, enables precisely deposited layers with control of their thickness at the atomic scale. The ALD method enables the deposition of highly uniform and conformal layers, even on complex geometries and within surface defects such as pores or pinholes. Due to these unique properties, the ALD is preferred for biomedical applications, where it can effectively seal structural discontinuities in PVD coatings. For example, the work [17] used a combination of two CrN layers produced by magnetron sputtering, between which an Al_2_O_3_ coating was applied using the ALD technique. The authors of the paper [17] demonstrated, based on corrosion tests, that the use of an interlayer, produced by the ALD process, allows a significant increase in the effectiveness of the corrosion protection of the coating—an increase in the coefficient of corrosion protection effectiveness of the coating was obtained from 50% for the CrN coating to 90% in the case of a hybrid CrN/Al_2_O_3_/CrN coating. In the publication [18], the positive effect of the interlayer obtained by the ALD process on the mechanical properties of the PVD-deposited coating was also shown. In this case, the use of two, three, and four Al_2_O_3_ interlayers, respectively, applied by the ALD process between successive layers of CrAlSiN coating obtained by reactive magnetron sputtering on a stainless steel substrate was compared. The grinding of the PVD coating grain was found to be responsible for improving the mechanical properties of the coating; furthermore, as the number of Al_2_O_3_ interlayers increased, the effect was enhanced. In this work [18], using two Al_2_O_3_ interlayers, a higher efficiency was also achieved to improve the anticorrosion properties of the CrAlSiN coating—the corrosion current density decreased from 9.41 × 10^−6^ to 2.54 × 10^−9^ µA/cm^2^.

However, the authors in the article [19] investigated the effect of a 4 nm thick Al_2_O_3_ layer applied by the ALD process on the corrosion resistance of TiN/TiAlN and Ti(C,N) coatings applied by cathodic arc evaporation in the PVD process on a tool steel substrate. They showed a significant improvement in the corrosion resistance obtained in potentiodynamic studies (where a decrease in the density of the corrosion current was observed, to values of the order of 10^−7^ A/cm^2^) [19]. On the other hand, in the work [20], improvements in the corrosion properties of hard CrN and diamond-like carbon (DLC) coatings deposited on low-alloy steel were achieved by sealing with 50 nm thick ALD layers consisting of Al_2_O_3_ and Ta_2_O_5_ nanolaminates. The authors demonstrated that the Al_2_O_3_ and Ta_2_O_5_ layers deposited by ALD conformally followed the surface topography of the CrN coatings. Moreover, ALD deposition was observed to extend into the pinholes present in the CrN layer. Electrochemical studies demonstrated improved corrosion resistance of CrN-coated steel, attributed to effective sealing of the coating structure, resulting in a reduction in the corrosion current density. In parallel, the resistance under neutral salt spray (NSS) conditions was significantly enhanced, with the onset of visible corrosion defects postponed from 2 h to 168 h. When DLC coatings were applied, a marked improvement in NSS durability was also achieved, clearly indicating the sealing of structural defects [20]. Furthermore, in several of their own studies [21,22,23,24,25,26] on this topic, including, in particular, the work [21], it was shown that the use of a treatment that combined PVD and ALD processes allows a significant improvement in corrosion resistance. In this case, a reactive magnetron sputtering method (PVD technique) was combined with an ALD process, in which a TiO_2_ coating was produced on a 316L steel substrate. In a potentiodynamic test conducted in a 3.5% NaCl solution, there was an eight-fold decrease in the corrosion current density values compared to uncoated steel.

**Table 1:** Performance comparison of selected inorganic coatings deposited on 316L stainless steel substrates [1,2,3,4,6,7,8,11,13,14,15,17,18,21,22,27,28,29,30,31,32].

Coating	Deposition Method	Key Advantages	Main Limitations	Typical Applications
TiN	PVD	High hardness, good wear, and corrosion resistance	Limited oxidation resistance at high temperature	Biomedical tools, tribological coatings
CrN	PVD	Excellent corrosion resistance, strong adhesion	Slightly lower hardness than TiN	Marine, chemical, and energy sectors
Al_2_O_3_	CVD (ALD), sol–gel, plasma spray	Outstanding thermal and chemical stability	Brittleness, moderate adhesion to steel	High-temperature corrosion barriers
ZrO_2_	Sol–gel, PVD (ALD), plasma spray	High thermal stability, good biocompatibility	Moderate wear resistance	Biomedical implants, thermal barrier coatings
SiO_2_/TiO_2_	Sol–gel, PECVD, ALD, PVD	Good corrosion protection, biocompatibility	Limited wear resistance	Protective and functional surface layers
Cr_2_O_3_	Thermal oxidation, plasma spray	Excellent wear and corrosion resistance	Limited biocompatibility	Severe wear and corrosive environments
Hydroxyapatite (HA)	Plasma spray, sol–gel	Excellent biocompatibility and bioactivity	Low mechanical strength	Biomedical implant coatings

The proposed publication is a continuation of research on the effect of the titanium oxide deposition conditions of ALD on the structure and properties of CrN/TiO_2_ coatings obtained by the PVD/ALD hybrid method. In an earlier work [26], the effect of this hybrid coating on the performance properties deposited on Al-Si-Cu aluminium alloy substrates was studied. This time, this paper will present the results of structural studies and mechanical and corrosion properties of the PVD/ALD CrN/TiO_2_ hybrid coating deposited on a 316L austenitic steel substrate according to the number of 200 to 1000 ALD TiO_2_ layer application cycles.

## 2. Materials and Methods

### 2.1. Materials

The tests were performed on austenitic Cr-Ni-Mo (316L) type steel (EN mark: X5CrNiMo17-12-2; number: 1.4401) coated with coatings obtained by a hybrid process combining PVD and ALD methods. A steel rod with a diameter of Φ = 14 mm was sectioned into discs with a thickness of g = 5 mm. Then, these discs were subjected to mechanical finishing, including grinding and polishing, to achieve a surface roughness of Ra ≤ 0.02 μm. Before coating, the substrates were cleaned in ultrasonic baths using acetone. CrN coatings were produced using the PVD technique, after which TiO_2_ layers were deposited by ALD in three different thickness variants corresponding to 200, 500, and 1000 deposition cycles.

The conditions of the MS-PVD process are shown in Table 2. Table 3 shows the synthesis conditions of the ALD process.

The reference materials were uncoated substrates and those coated with CrN coating made by the PVD process (without ALD modification).

### 2.2. Methods

A Zeiss Supra 35 (Zeiss, Oberkochen, Germany) scanning electron microscope, equipped with an EDS spectrometer (for chemical analysis in microareas), was used to evaluate the coating structure and surface morphology.

The coating morphology was analysed by atomic force microscopy type Park Systems XE-100 AFM (Suwon, Republic of Korea). Non-contact mode was used, and the measuring areas were 10 × 10 μm and 1 × 1 μm.

X-ray diffraction measurements were performed using a PANalytical X’Pert Pro MPD diffractometer (PANalytical, Almelo, The Netherlands). The analysis employed filtered radiation from an X-ray tube equipped with a cobalt anode (Co Kα = 0.179 nm) and an iron filter, operating at 40 kV and a filament current of 30 mA. The measurements were conducted in the Bragg–Brentano geometry over an angular range of 30–110° (2θ), with a step size of 0.05° and a counting time of 100 s per step.

To ensure accurate identification of the coating, Grazing-Incidence X-ray Diffraction (GIXRD) geometry was additionally applied. The obtained diffraction data were analysed by means of the X’Pert HighScore Plus software (v. 3.0e) with a dedicated Inorganic Crystal Structure Database—ICSD (FIZ, Karlsruhe, Germany).

The electrochemical properties of the studied hybrid coatings were examined in 3.5% NaCl solution at room temperature via a potentiodynamic test and electrochemical impedance spectroscopy (EIS) method using an Atlas 0531 EU potentiostat (ATLAS-SOLLICH, Rębiechowo, Poland). The Ag/AgCl electrode was used as the reference electrode, and a platinum rod was used as the counter electrode. First, the open circuit potential (Eocp) for 1 h was measured, and then from the initial potential E_star_t = E_ocp_ − 100 mV to E_finish_ = 1 V or a current density of 1 mA/cm^2^, the rate of potential increase was 1 mV/s.

Characteristic electrical quantities that describe corrosion resistance, namely current density (J_corr_), corrosion potential (E_corr_), and polarisation resistance (R_pol_), were determined using the Tafel method and AtlasLab software (Version 2.24, Atlas Sollich, Banino, Poland).

Electrochemical impedance spectroscopy (EIS) was also performed to further understand the corrosion protection of the study’s coating, using a frequency range of 100 kHz to 10 mHz and an amplitude of 10 mV. The data obtained are illustrated using Nyquist and Bode representations. An equivalent circuit was created to model the electrochemical processes, with numerical fits from AtlasLab and EC-Lab, including CPEs to capture non-ideal capacitance.

The contact angle measurements were conducted using a Biolin Scientific Attension Theta Flex optical tensiometer (Espoo, Finland), by the sitting drop method. Distilled water and diiodomethane served as the test liquids, with a standardised droplet volume of 2 μL applied to each.

Wetting angle measurements were made as a function of time (60 s) in a series of five measurements for each sample. The measurements were carried out at a room temperature of 289 K (25 °C).

Wear resistance tests were performed using the CSM Instruments tribometer (Anton Paar, Peseux, Switzerland) in using the “pin-on-disc” method. The test was carried out in the reciprocating mode. A 6 mm diameter WC-Co ball was used as a countersample. The test parameters were as follows: load = 5 N, number of cycles = 20,000, abrasion radius = 3 mm, speed = 5 cm/s. The friction coefficient was recorded as a function of the number of cycles during the test. Post-test wear track geometries were analysed using a Taylor–Hobson Surtronic 25 contact profilometer. Wear rate defining the wear rate of the material due to abrasion, calculated from the relationship: wear rate = V/(F_s_) [mm^3^/Nm], where V—volume of worn material (mm^3^), F—contact force (N), s—friction path (m).

The adhesion of the coating to the substrate was assessed using the Anton Paar Revetest RST scratch tester (Corcelles-Cormondrèche, Switzerland). A progressive load of 0 to 100 N was applied, and the measurement path length was 10 mm. The critical load L_c_, at which the adhesion of the coating was lost, was determined by the acoustic emission value recorded during the measurement and observation of the scratches created during the test.

## 3. Results

**Figure 1:**
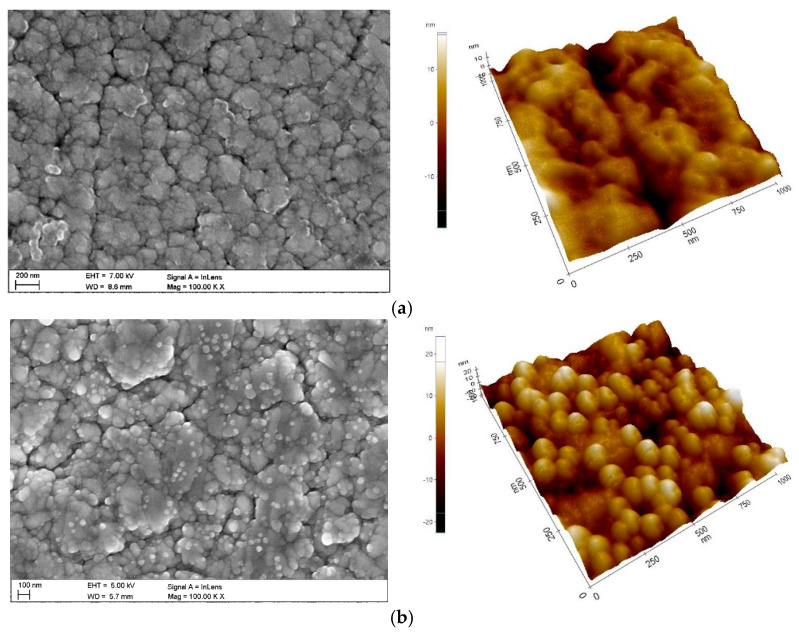
Morphology of PVD/ALD coatings (SEM, AFM): (**a**) CrN/TiO_2_(500), (**b**) CrN/TiO_2_(1000).

The microscale topography of hybrid coatings shows typical coatings produced by the MSPVD method, as shown in Figure 1 (SEM images). The coatings are characterised by morphological imperfections, such as small pores and microcracks, as well as surface scratches that remain after mechanical preparation of the substrate for coating. EDS analysis confirmed the presence of elements consistent with the tested coatings, in particular Cr, Ti, O, and N.

At the nanoscale, the morphology of the hybrid coatings depends on the number of cycles in the ALD process (Figure 1). Both CrN/TiO_2_(200) and CrN/TiO_2_(500) coatings show a wavy surface similar to the CrN coating, indicating that the ALD layer formed at a lower number of cycles is uniform and accurately reflects the topography of the PVD coating. The CrN/TiO_2_(1000) coating shows a granular surface morphology throughout the entire area tested. Table 4 shows the results of the surface roughness obtained by AFM analysis.

**Figure 2:**
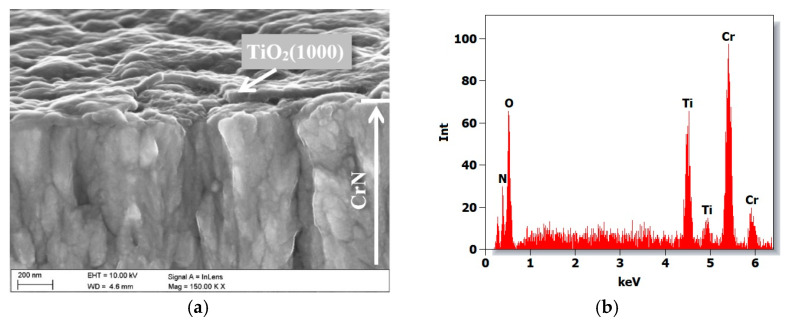
Microstructure of the CrN/TiO_2_(1000) hybrid coating: (**a**) fracture image (SEM), (**b**) EDS spectrum of the area as in (**a**).

The microscopic analysis of the fractures revealed that the layers deposited by the ALD method adhere tightly to the PVD base, which remains well-bonded to the substrate. The PVD coatings display a columnar, fibrous structure typical of the Thornton model’s T-zone [33]. As expected for PVD/ALD hybrid coatings, the titanium oxide layer follows the PVD topography evenly, effectively eliminating structural discontinuities by filling inherent gaps in the coating (Figure 2).

**Figure 3:**
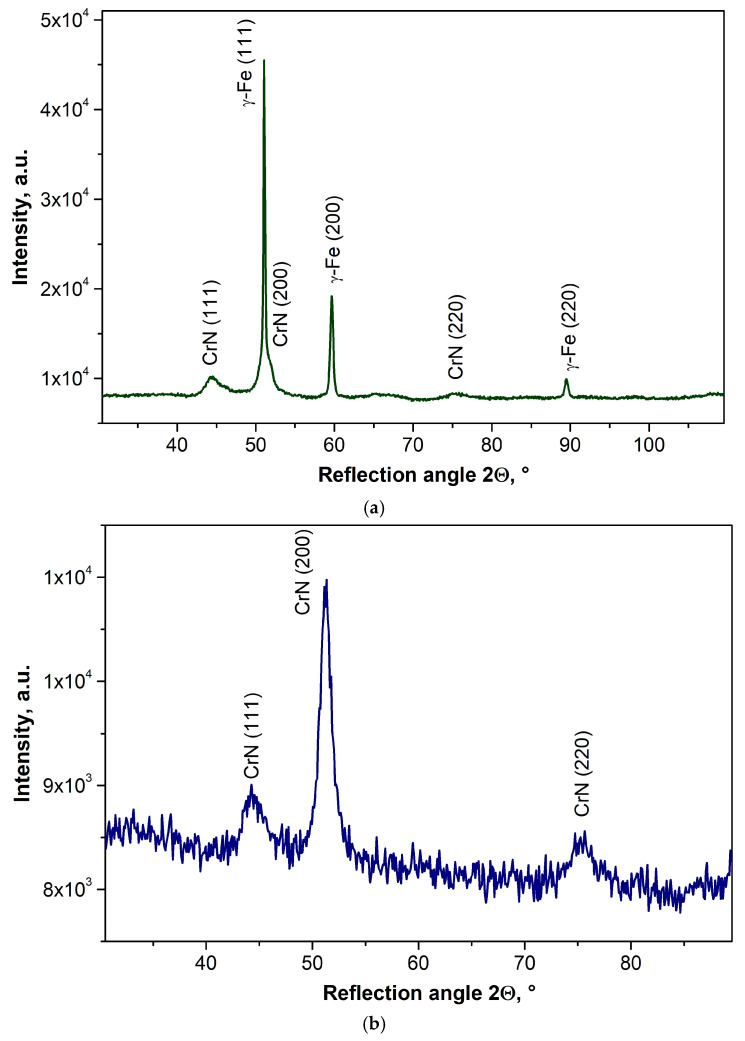
X-ray diffraction pattern of CrN/TiO_2_(500) coating obtained by (**a**) Bragg–Brentano method, (**b**) GIXRD method (α = 1.5°).

A qualitative analysis of the phase composition carried out using X-ray diffraction confirms that a coating containing the CrN phase (crystal system: cubic, F m −3 m, ICSD reference code: 98-015-2809) was successfully formed on a 316L austenitic steel substrate (Figure 3a). X-ray diffraction patterns obtained using the Bragg–Brentano technique also revealed the presence of reflections from the γ-Fe phase (crystal system: cubic, F m −3 m, ICSD reference code: 98-018-5720) present in the substrate material. The presence of reflections from the substrate is due to the thickness of the coating obtained (g ≈ 2 µm), which is less than the penetration depth of the X-rays into the material. As a result of investigations using the grass-incidence X-ray diffraction (GIXRD) technique at low angles of incidence of the primary X-ray beam, reflections were recorded exclusively from thin surface layers (Figure 3b). The absence of reflections from phases present in the substrates on the diffraction patterns obtained using the GIXRD technique indicates that the X-ray beam penetrating the tested coatings did not reach the substrate. It should be noted that in the case of CrN/TiO_2_ hybrid coatings, the presence of a crystalline titanium oxide phase was not confirmed. This is undoubtedly due to the fact that in the layers obtained after 200 and 500 ALD cycles, the crystalline titanium oxide phase is absent. It is true that in the titanium oxide layers obtained after 1000 ALD cycles, a crystalline phase of rutile grains is already present in an amorphous matrix, as confirmed by studies [26,34] and microscopic morphological studies (Figure 1), the thickness of these layers does not allow the presence of the crystalline phase of titanium oxide due to the detection threshold of the XRD method.

**Figure 4:**
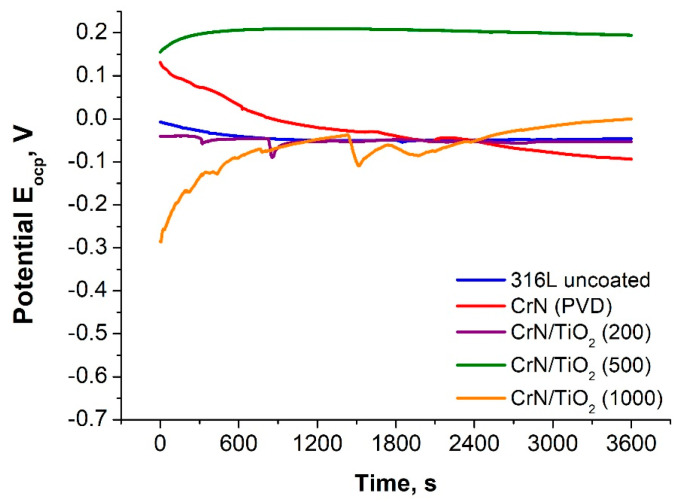
Open-circuit potential for uncoated and coated 316L steel.

**Figure 5:**
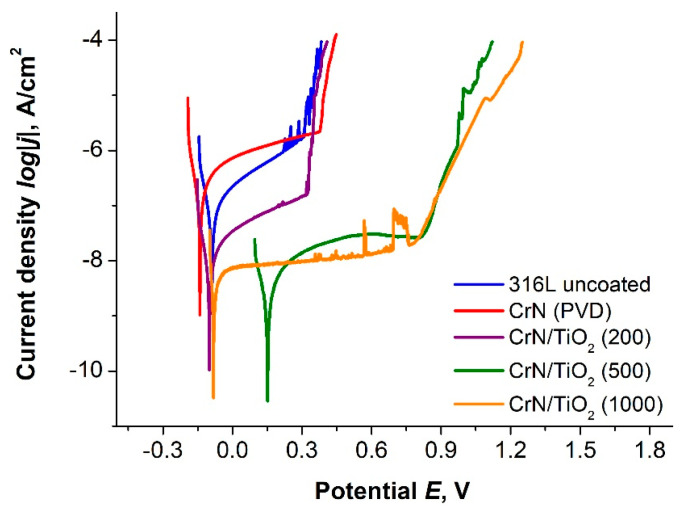
Potentiodynamic polarisation curves for uncoated and coated 316L steel.

The first stage of corrosion resistance testing consisted of recording the potential of the stationary circuit under current-free conditions for 1 h (Figure 4). In the next stage, potentiodynamic polarisation curves in the cathodic and anodic ranges were recorded for the Tafel analysis (Figure 5), the results of which are shown in Table 5.

The value of the E_ocp_ potential for the sample with CrN coating applied by the PVD process was lower than that of the 316L steel substrate material, while the application of hybrid coatings, after 500 and 1000 cycles, resulted in a higher potential value. In the case of the CrN/TiO_2_(200) sample, the difference in the stationary potential value with respect to the steel substrate sample was very small (7 mV), so it can be concluded that in a solution of 3.5% NaCl, without forced current flow, the materials were in equilibrium at the same potential value.

The included current flow through the set test system and the application of the Tafel extrapolation method allowed recording the characteristic electrochemical values of the material: corrosion potential, corrosion current density, and polarisation resistance. When comparing the effect of the hybrid protective coatings, there was a very pronounced reduction in the obtained values of the corrosion current density relative to the substrate material, and similarly for samples with coatings exclusively obtained by the ALD process, there was an improvement in the values of the corrosion current density from 28.5 nA/cm^2^ to 0.8–0.9 nA/cm^2^ in favour of materials with CrN/TiO_2_ coatings. Confirmation of the difference in the change in resistance to the corrosive environment was found in the tested materials by comparing the values of polarisation resistance, which took the highest values for the coatings obtained by the hybrid process, and the lowest value was obtained by the material with CrN coating. The highest change in polarisation resistance was found for hybrid samples after 500 application cycles (10,640 kΩ∙cm^2^) and the sample after 1000 cycles (4117 kΩ∙cm^2^), with the CrN/TiO_2_ sample also showing a significant increase in the R_pol_ value of 10 times that of 338 kΩ∙cm^2^ for uncoated 316L steel.

The hybrid process coatings applied to the steel substrate contributed to very good protection of the substrate against pitting damage, as evidenced by an increase in the pitting initiation potential (E_b_) from 394 mV to more than 1000 mV for the coatings after 200, 500, and 1000 application cycles. A trend of increasing the potential for E_b_ was also observed with an increasing number of cycles of CrN/TiO_2_ coatings applied.

When comparing the values of the determined corrosion potential of the tested samples, the description related to the open circuit potential can be used for their interpretation, as the same relations and their highest value were observed here for the sample with CrN/TiO_2_(500) coating.

**Figure 6:**
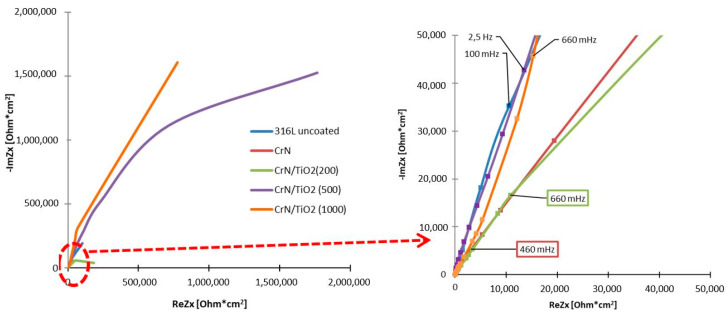
Nyquist impedance diagram for uncoated 316L steel coated with CrN/TiO_2_ hybrid coatings.

**Figure 7:**
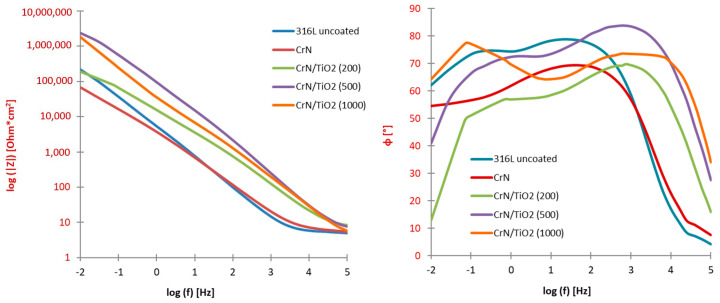
Bode impedance diagram for uncoated 316L steel coated with CrN/TiO_2_ hybrid coatings.

**Figure 8:**
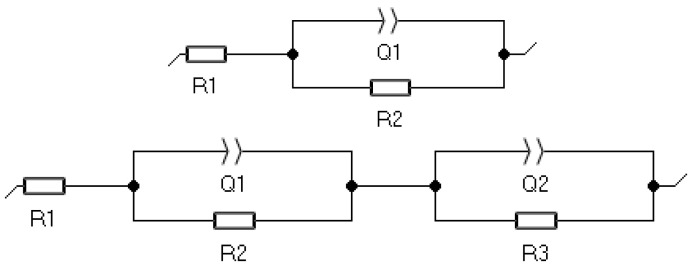
An equivalent circuit that represents the impedance spectra.

The EIS was employed to characterise the electrochemical properties of both single PVD and hybrid PVD + ALD coatings. Based on the recorded impedance spectra, presented in the form of Nyquist (Figure 6) and Bode (Figure 7) plots, appropriate equivalent electrical circuits were fitted to the experimental data to represent the corrosion systems studied. In the case of the substrate material sample and the CrN-coated sample, the circuit consisted of a single loop, and in the case of the hybrid-coated samples, the circuit consisted of two loops connected in series, each made up of a CPE constant phase element and a resistor (Figure 8), and its resultant impedance can be written with the following relations:


$$Z=R1+\frac{1}{\frac{1}{R2}+{Y_{1}(j\omega )}^{\alpha 1}}\;\;Z=R1+\frac{1}{\frac{1}{R2}+{Y_{1}(j\omega )}^{\alpha 1}}+\frac{1}{\frac{1}{R3}+{Y_{2}(j\omega )}^{\alpha 2}}$$

In the proposed electrical circuit, R1 is related to the resistance of the electrolyte, i.e., the 3.5% NaCl solution, R2 represents the resistance of charge transfer to the electrolyte from the surface zone of the material, i.e., the first coating in the electrolyte, in this case the PVD coating, the CPE2 element models the capacitance of this zone, R3 is the resistance of charge transfer across the phase boundary, and the CPE3 element can be considered as a reflection of the electrical properties of the double layer at the phase boundary, the values of these elements are summarised in Table 6.

Analysing the behaviour of the curves in the Nyquist plot, it can be concluded that the highest resistance to corrosion of the tested environment was obtained by the sample with hybrid coating after 500 and 1000 cycles, for which the highest value of the angle of inclination to the OX axis was identified. When registering the impedance spectrum at high frequencies, it is also evident that the corrosion damage rate is significantly reduced for the samples with PVD + ALD coatings after 500 and 1000 application cycles, compared to the 316L substrate material.

The response of the corrosion system across a broad frequency range was analysed using Bode plots (Figure 7). Monitoring the impedance revealed the expected trend: the impedance decreased as the voltage signal frequency increased.

By recording changes in the impedance of the tested samples, a typical behaviour of curves was found, i.e., decreasing impedance values with increasing frequency of the voltage signal.

The lowest impedance value was found in the entire range of frequencies tested for samples of the substrate material and with the CrN coating applied, while the highest impedance value was recorded for the sample with the hybrid coating after 500 application cycles.

When comparing the impedance values for the 316L steel substrate material with the sample with the coating after the PVD process, it is possible to distinguish the frequency range in which the uncoated sample was characterised by its lower value (from 100 kHz to 40 Hz) and the range from 25 Hz to 10 mHz in which the lower impedance was found for the sample with the CrN coating.

Analysis of the second Bode plot, showing the dependence of the phase shift angle on the impedance modulus, revealed that the highest phase angle value, exceeding 80°, was observed for the sample with the hybrid coating deposited using 500 cycles, in the frequency range from 100 Hz to 2.5 kHz.

For all samples, high values of phase shift angle >60° were recorded in a wide frequency range from 1 Hz to 1 kHz, with very high frequency values for the uncoated and CrN coated sample. The value of this angle quickly decreased below 30 degrees and less, while the better quality was characterised by the CrN/TiO_2_(200) coated sample.

Based on contact angle measurements, uncoated 316L stainless steel exhibited a hydrophilic surface character. After the application of the coating, all modified samples showed a clear transition to hydrophobic behaviour. This indicates that the surface modification effectively altered the wettability of the material (Table 7).

For the CrN/TiO_2_(500) sample group only, the wetting angle values fluctuated around the threshold between hydrophilic and hydrophobic states. At the same time, the coating of chromium nitride with a titanium oxide layer obtained by the PVD/ALD process causes a change in the wetting angle in the direction of hydrophobicity. In this case, the increase in hydrophobicity can be associated with an increase in nanoroughness, which leads to the formation of areas with air bubbles under the water droplets. This mechanism explains the so-called Cassie–Baxter condition [18]. The wetting angle θ with diiodomethane varies from 44° ± 3° to 69° ± 1°. Surface free energy values are shown in Table 7. There is a significant disproportion between the dispersive and polar components, i.e., γdS ≫ γpS, for all coated samples studied. The surfaces have affinity for nonpolar SFE groups.

The abrasion resistance test showed a clear impact of the proposed PVD/ALD hybrid coating system on the tribological wear of 316L austenitic steel. The highest “wear rate” was recorded for the CrN/TiO_2_ hybrid coating produced with the lowest number of ALD cycles, for which the wear volume was in the sensitivity limit of the measurement method. The CrN coating alone significantly reduced surface degradation compared to the substrate, indicating effective transfer of contact loads by the chromium nitride layer and reduction in plastic deformation of the base material (Table 8).

However, increasing the number of ALD cycles did not lead to a systematic improvement in tribological properties. For the CrN/TiO_2_ system obtained at 1000 cycles, the highest wear intensity was observed among all variants, exceeding even that of the 316L steel. This means that an excessively developed TiO_2_ layer loses its ability to work under contact load. Its brittleness led to the initiation of microcracks and spalling, and the resulting wear particles acted as an abrasive agent, intensifying the microgrooving of the surface.

**Figure 9:**
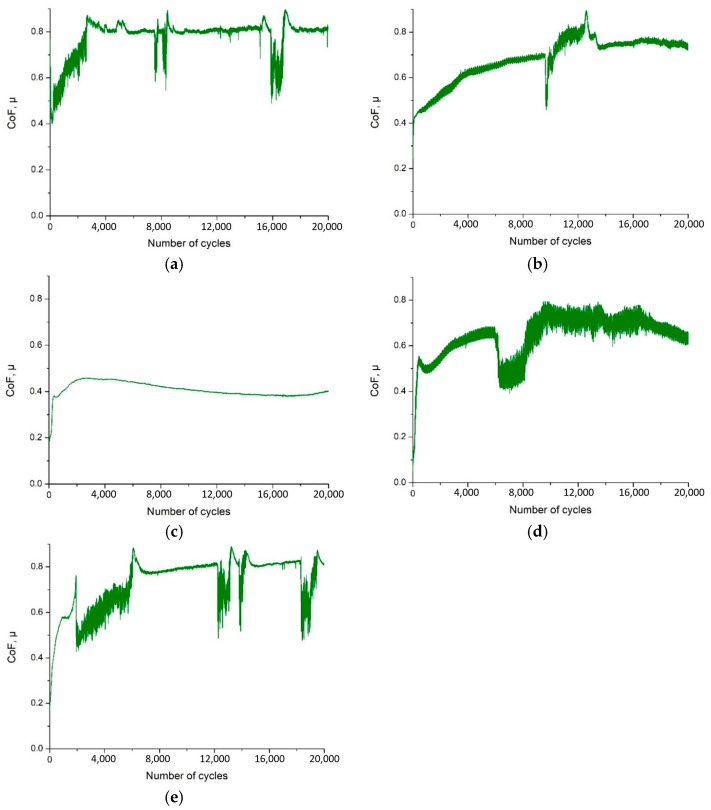
Friction coefficient graphs: (**a**) 316L uncoated, (**b**) CrN coating, (**c**) CrN/TiO_2_(200) coating, (**d**) CrN/TiO_2_(500) coating, (**e**) CrN/TiO_2_(1000) coating.

The friction coefficient µ(t) curves confirm the wear observations during the tests (Figure 9). For uncoated 316L steel, the friction coefficient stabilised almost from the beginning of the test at 0.78 to 0.80 and remained stable for 20,000 cycles (Figure 9a). Initially, the friction coefficient increased from approximately 0.45 to 0.75–0.85, corresponding to the running-in phase and direct ceramic-metal contact. In the second part of the test, there were short-term decreases of 0.5 to 0.6 associated with the detachment of adhesive deposits. Such an unstable course indicates the dominance of adhesive wear and intense plastic deformation of the surface.

The application of CrN coating on the tested substrate reduced the values of the friction coefficient and stabilised its course by limiting adhesion. The local increase in µ in the final phase of the test resulted from the local abrasion of the coating and exposure of the substrate, after which the behaviour of the system temporarily resembled that of uncoated steel (Figure 9b).

The most stable performance was obtained for a thin CrN/TiO_2_ layer: the average µ value was approximately 0.41 throughout the range of cycles (Figure 9c). The absence of sudden jumps indicates that continuous contact between the ceramic and the countersample was maintained without coating breakage. For other variants, µ values of approximately 0.64 to 6000–7000 cycles and approximately 0.59 to approximately 6000 cycles were observed, followed by an increase in instability associated with layer damage. In the case of a thicker TiO_2_ coating (approximately 0.75 to approx. 9000 cycles), rapid fluctuations in µ occurred, indicating cyclic cracking and exposure of the substrate (Figure 9d,e).

**Figure 10:**
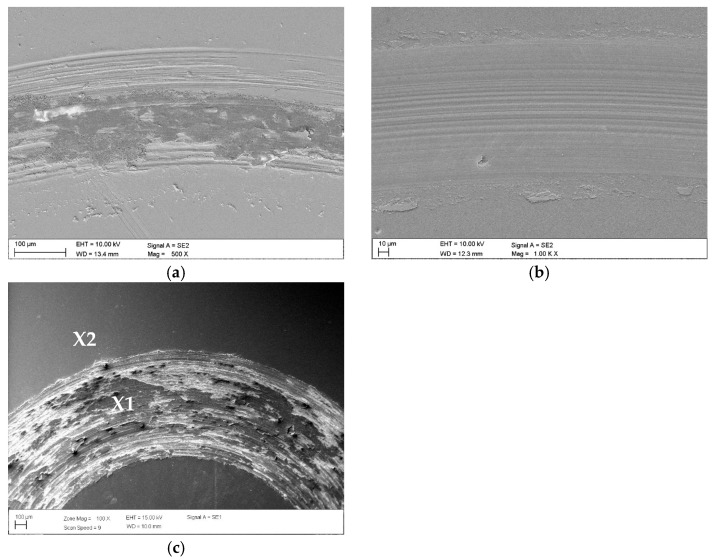
Wear marks: (**a**) uncoated 316L, (**b**) CrN/TiO_2_(200) coating, (**c**) CrN/TiO_2_(1000) coating.

**Figure 11:**
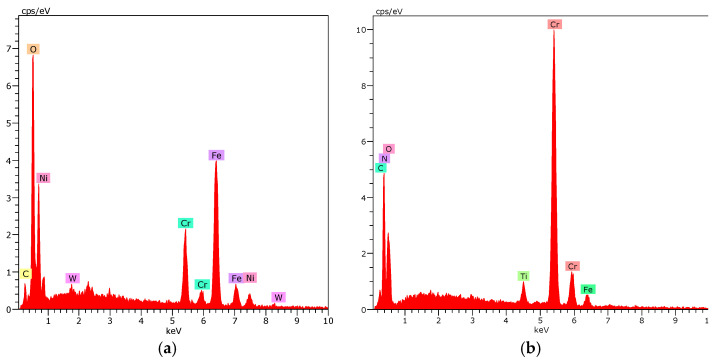
X-ray energy dispersive plot as in Figure 10c: (**a**) the area X1, (**b**) the area X2.

The morphology of the wear marks was consistent with the friction coefficient curves (Figure 10). For 316L steel, a wide friction track, furrows parallel to the direction of movement, material flow, and the decrement characteristic of adhesive wear and delamination were observed (Figure 10a). The use of a hybrid coating with a low number of cycles resulted in a narrow and uniform wear mark with mild oxide abrasion, without deep furrows and detachments (Figure 9b). In contrast, for coatings with a higher number of ALD CrN/TiO_2_ cycles (1000), cracks, spalling, and local delamination were visible, and detached fragments of the coating caused abrasive wear (Figure 10c and Figure 11).

The results of adhesion tests using the scratch test method also confirm a change in the degradation mechanism of the tested coating system. For CrN coating applied to a 316L steel substrate, the first damage threshold occurred at Lc_1_ ≈ 9 N, crack propagation was observed at Lc_2_ ≈ 18 N, and substrate exposure occurred at Lc_3_ ≈ 22 N, indicating the progressive nature of the damage and good adhesion. In PVD/ALD CrN/TiO_2_ hybrid coating systems, critical threshold values decreased with increasing TiO_2_ layer thickness (Lc_1_: from 11.5 to 7.6 N; Lc_2_: from 16.0 to 11.5 N; Lc_3_: from 17.3 to 16.5 N), indicating earlier brittle fracture and delamination at the interlayer boundary (Table 9).

**Figure 12:**
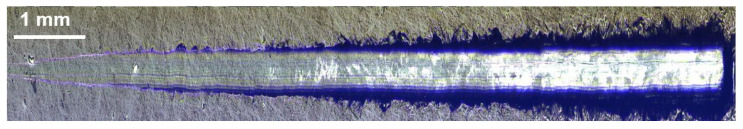
Scratch formed after the adhesion test in the CrN coating applied to the 316 L austenitic steel substrate.

**Figure 13:**
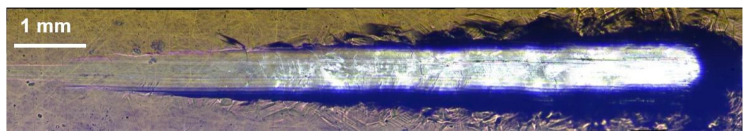
Scratch formed after the adhesion test in the CrN/TiO_2_(500) hybrid coating applied to the 316 L austenitic steel substrate.

Analysis of the morphology of the marks left after the adhesion test showed that the degradation of both coating systems proceeded gradually, but the destruction mechanism was strongly dependent on the layered structure. In the initial load range, regardless of the type of coating, micro-cutting and local plastic deformation dominated. This manifested itself in the formation of a narrow and relatively homogeneous scratch with parallel furrows consistent with the direction of movement of the indenter. The lack of clear delamination indicates that the shear stresses generated by contact did not yet exceed the adhesive strength of the coating-substrate interface (Figure 12 and Figure 13). In the PVD-produced case of CrN coating, with increasing load, there was a gradual widening of the scratch track and the initiation of microcracks, mainly along the columnar microstructure characteristic of this deposition technology (Figure 12). The damage developed in a dispersed manner, and the 316L steel substrate was exposed only in the final area of the mark. This pattern indicates relatively good coating adhesion and the ability of the system to relax stresses through local microcracking and partial plastic deformation, which delays rapid delamination. Different tribological wear mechanisms were observed in the CrN/TiO_2_ (PVD/ALD) hybrid system. In this case, the increase in load caused an earlier loss of continuity of the surface layer. In this case, the increase in load caused an earlier loss of continuity of the top layer. The scratch marks were larger, with irregular edges, numerous chips, and extensive delamination zones. The degradation mechanism was mixed: it included brittle cracking of the TiO_2_ layer and delamination at the interlayer boundary (Figure 13). The compact and rigid layer deposited by the ALD method limits the possibility of dissipation of deformation energy, leading to a concentration of contact stresses and a more violent destruction. In summary, PVD CrN coating exhibits a more gradual progression of quasiductile damage, while the CrN/TiO_2_ hybrid coating system is more susceptible to brittle cracking and adhesive delamination at higher loads. These differences result from the different mechanical properties and microstructure of the layers, which determine how stresses accumulate and dissipate during the scratch test.

The phenomena observed during the abrasion resistance and adhesion tests can be linked to changes in the structure of TiO_2,_ depending on the number of ALD cycles. At low thickness (200 cycles), the layer is amorphous and conformally seals CrN defects, limiting stress concentration. As the number of ALD cycles increases (500–1000), partial crystallisation (anatase/rutile) occurs, which increases stiffness and brittleness, leading to the initiation of microcracks in the high-stress point contact area. The results obtained correlate directly with observations on wear resistance and critical L_c_ values: an optimal thin CrN/TiO_2_ coating simultaneously minimises the friction coefficient, reduces microcracks, and maintains high adhesion, while an excessively thick TiO_2_ layer impairs both the adhesion and tribological durability of the system.

## 4. Summary

**Figure 14:**
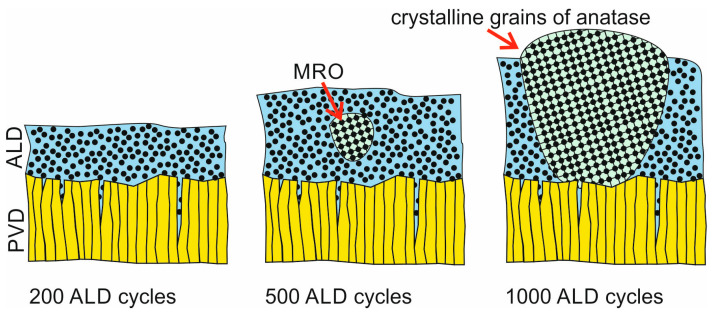
The structure of the TiO_2_ coating depends on the number of ALD cycles.

The microstructure of the titanium oxide layers depends on the number of cycles in the ALD process. With fewer cycles, the resulting layer is amorphous, with areas of medium-range order (MRO), which are the nuclei of the anatase crystalline phase. The crystallisation of TiO_2_ occurs in two main stages: nucleation in the amorphous matrix and grain growth (Figure 14). The driving force behind the phase transformations during film growth is the difference in free energy between the structural components of titanium oxide. Basically, these transformations follow the Ostwald–Lussac rule [35,36,37], going from the amorphous phase to anatase and eventually to rutile as the most stable phase. According to this rule, anatase is a transition phase, less stable compared to rutile. Therefore, it can be expected that as the number of ALD cycles or the higher deposition temperatures increase, the rutile phase forms because of its greater stability, gradually replacing anatase [26,28,34].

Comparison of the results of corrosion testing of coated products allows us to conclude that the best corrosion properties are provided by hybrid coatings consisting of a PVD layer and an additional applied ALD layer, as evidenced by a reduced corrosion current value of more than 30 times with respect to uncoated 316L steel and an increase in the polarisation resistance value by one order of magnitude. The best results were obtained for the sample after 500 cycles of layer deposition, for which the lowest current density was obtained, with a change in the open circuit potential and corrosion potential by more than 230 mV, to positive values. The CrN/TiO_2_(500) coated material showed the highest increase in polarisation resistance with respect to the uncoated sample, up to 31 times, while the favourable increase in polarisation resistance by more than 10 times should also be noted for the other two variants of hybrid coated samples. The confirmation of the results obtained, that is, a significant improvement in the corrosion properties of the hybrid sample after 500 application cycles, was obtained in impedance spectroscopy studies. The record recorded a significantly higher impedance value throughout the frequency range and a higher phase change angle than for uncoated 316L steel, indicating better quality or tightness of the obtained coating [38]. No improvement in resistance to the applied corrosive environment was found in the case of the PVD-applied CrN-coated sample, where virtually all parameters were worse than those of the uncoated steel.

According to the current state of knowledge, there is a correlation between wettability, surface roughness, and corrosion resistance [39,40]. In general, surface wettability is influenced by the chemical composition of the surface, the free energy, the surface morphology, and the properties of the measuring liquid. Hydrophobicity or hydrophilicity, with a general consideration of the wetting state (Wenzel, Cassie–Baxter, or intermediate state), will affect the presence of water on the surface, and thus the degree of corrosion of the material under test. In general terms, the hydrophobicity of a surface promotes greater corrosion resistance. However, this assumption does not take into account the characteristics of the surface topography, including its microscale and nanoscale roughness. Reduced roughness can promote better corrosion resistance, as proven by atomic force microscope roughness measurements and corrosion resistance tests, which is evident for samples with a hybrid coating applied. A similar relationship is also presented in the paper [23]. In truth, for samples with coatings, the recorded values of wetting angles are significantly higher than those represented by the material in the initial state, but the electrochemical properties are more influenced by the type of material surface than by the geometric state of the surface. The stability of the surface and/or surface layer should be considered here. In the case of austenitic steels 316L, the oxide layer formed on the surface in a spontaneous manner is native in nature, and aggressive ions of corrosive solutions easily penetrate it, leading to an increase in the corrosion rate. As for the hybrid coatings produced, they exhibit good stability and poor ion permeability, which explains the improved corrosion resistance. Studies of the barrier properties of titanium oxide (ALD) for ion diffusion are presented in the paper [41,42]. In turn, the recorded values of the wetting angle are probably due to the wetting condition. For hybrid-coated samples, a wetting state consistent with the Wenzel or intermediate state is likely, where the liquid/solid interface line is characterised entirely by the liquid/solid surface tension or intermediate liquid/air/solid. It should be noted that the differences in the recorded values for the samples tested were similar, and evaluating the physicochemical properties is a difficult and very complex process.

Mechanical property tests showed that the tribological durability of the coating system does not depend solely on the thickness of the top layer but rather on the complex interaction between the material structure of the coating, its adhesion to the substrate, and the wear mechanisms. The highest wear resistance was observed for a thin hybrid CrN/TiO_2_(200) coating produced with a minimum number of ALD cycles, indicating the key role of maintaining continuous and stable ceramic-metal contact without initiating microcracks.

Analysis of the friction coefficient and the morphology of the wear pattern suggests that the effectiveness of surface protection results from the synergy of load-bearing and adhesion mechanisms. The CrN coating effectively absorbs contact loads, reducing plastic deformation of the substrate, while the thin TiO_2_ layer acts as a sealing layer for CrN defects, improving local interlayer adhesion and reducing stress concentration. An increase in TiO_2_ thickness leads to partial crystallisation of the layer (anatase/rutile), which increases its stiffness and brittleness, promoting the initiation of microcracks and delamination, thus impairing both adhesion and tribological durability.

Scratch tests and measurements of critical Lc values confirm that with the appropriate coating thickness, progressive failure characteristics and high adhesion to the substrate are maintained, while excessive thickness of the top layer leads to premature brittle cracking and exposure of the base steel. These results emphasise the importance of optimising coating architecture to maximise both wear resistance and adhesive mechanisms that ensure durable adhesion of layers, which is consistent with the literature on ALD layers used on PVD coatings [21,27].

## 5. Conclusions

In summary, it should be stated that

- CrN/TiO_2_ hybrid coatings on 316L steel substrates significantly improve electrochemical properties compared to uncoated substrates and those coated by single PVD processes. The CrN/TiO_2_(500) coating shows the best resistance to pitting corrosion.
- The structure of TiO_2_ coatings is dependent on the deposition conditions, especially the number of ALD cycles. At 200 and 500 ALD cycles at 200 °C, an amorphous layer is formed, while after 1000 cycles, crystalline anatase grains appear in the amorphous matrix. The process of formation and development of the crystalline phase of titanium oxides follows the Ostwald–Lussac rule.

## Figures and Tables

**Table 2:** Deposition conditions of MS-PVD coating.

Type of Layer	Magnetron Power, W	BIAS, V	Gas Flow, sccm		Temperature, °C
			Ar	N_2_	
transition layer of Cr	300	−60	100	-	200
CrN	350		35	200	

**Table 3:** Deposition conditions of ALD coatings.

Temperature, °C	Reagent Feed Time, s		Cleaning Time, s		Number of ALD Cycles
	TiCl_4_	H_2_O	After TiCl_4_	After H_2_O	
200	0.1	0.1	5	5	200
					500
					1000

**Table 4:** Nano-scale roughness results (AFM).

Sample	316L Uncoated	CrN	CrN/TiO_2_(200)	CrN/TiO_2_(500)	CrN/TiO_2_(1000)
Nano roughness S_a_, nm	1.10	4.76	4.67	3.61	4.96

**Table 5:** Potentiodynamic polarisation parameters for samples in 3.5% NaCl solution.

Sample	E_ocp_, mV	E_b_, mV	j_corr_, nA/cm^2^	E_corr_, mV	R_pol_, kΩ∙cm^2^	P_e_, % *
316L uncoated	−46	+394	28.5	−88	338	-
CrN	−93	+428	26.9	−144	150	−125.3
CrN/TiO_2_(200)	−53	+1060	0.9	−109	3685	90.8
CrN/TiO_2_(500)	+194	+1162	0.8	+151	10,640	96.8
CrN/TiO_2_(1000)	−2	+1292	0.8	−82	4117	91.8

* P_e_: corrosion protection efficiency, calculated as $\left(1-\frac{R_{pol\;substrate}}{R_{pol\;coating}}\right)\times 100\%$.

**Table 6:** Electrochemical impedance spectroscopy parameters.

Sample	R1 [Ω]	C1 [µF/cm^2^]	α1	R2 [kΩ]	C2 [µF/cm^2^]	α2	R3 [kΩ]
316L uncoated	3.9	83.93	0.82	1050	-	-	-
CrN	5.3	14.12	0.69	360	-	-	-
CrN/TiO_2_(200)	8.1	18.86	0.95	165	9.14	0.75	35
CrN/TiO_2_(500)	7.2	1.03	0.87	3615	7.27	0.872	17
CrN/TiO_2_(1000)	4.6	2.36	0.89	6492	5.4	0.792	18

**Table 7:** Contact angle measurements and surface energy calculated by the Owens–Wendt method.

Sample	Wetting Angle (°)			Surface-Free Energy (mJ/m^2^)		
	Distilled Water	Diiodomethane	γpS		γdS	γS
316L uncoated	62 ± 3	51 ± 1	20.64		19.99	40.53
CrN	100 ± 1	69 ± 1	1.22		27.7	23.92
CrN/TiO_2_(200)	101 ± 1	44 ± 3	0.03		44.14	44.18
CrN/TiO_2_(500)	94 ± 2	54 ± 1	0.85		33.21	34.05
CrN/TiO_2_(1000)	102 ± 2	49 ± 1	0.01		40.42	40.43

**Table 8:** Summary of the average friction coefficient, wear volume, and wear rate values for the CrN coating modified with thin TiO_2_ layers deposited using the ALD method, with a number of cycles ranging from 200 to 1000.

Sample	Coefficient of Friction, µ	Volume of Consumption, mm^3^	Wear Rate mm^3^/Nm
316L uncoated	0.81	0.194	1.03 × 10^−4^
CrN	0.64	0.139	7.38 × 10^−5^
CrN/TiO_2_(200)	0.41	0.000	0.000
CrN/TiO_2_(500)	0.75	0.131	6.95 × 10^−5^
CrN/TiO_2_(1000)	0.59	0.264	1.40 × 10^−4^

**Table 9:** Summary of the average critical load (L_c_) values determined by the scratch test, used as a measure of coating–substrate adhesion for investigated coatings.

Sample	Critical Load L_c_ [N]		
	L_c1_	L_c2_	L_c3_
CrN	9.0	18.0	22.0
CrN/TiO_2_(200)	11,5	16.0	23.0
CrN/TiO_2_(500)	8.7	14.2	17.3
CrN/TiO_2_(1000)	7.6	11.5	16.5

## Data Availability

The raw data supporting the conclusions of this article will be made available by the authors on request.
